# A randomized controlled trial of an intervention to reduce stigma toward people with opioid use disorder among primary care clinicians

**DOI:** 10.1186/s13722-023-00366-1

**Published:** 2023-02-11

**Authors:** Stephanie A. Hooker, A. Lauren Crain, Amy B. LaFrance, Sheryl Kane, J. Konadu Fokuo, Gavin Bart, Rebecca C. Rossom

**Affiliations:** 1 Research and Evaluation Division, HealthPartners Institute, 8170 33rdAve S, Mail stop 21112R, Minneapolis, MN 55440 USA; 2 Department of Psychiatry, University of Illinois at Chicago, Chicago, IL USA; 3 Division of Addiction Medicine, Hennepin Healthcare, Minneapolis, MN USA

**Keywords:** Healthcare providers, Substance use disorders, Stigma, Behavioral intervention, Patient narratives

## Abstract

**Background:**

Many primary care clinicians (PCCs) hold stigma toward people with opioid use disorder (OUD), which may be a barrier to care. Few interventions exist to address PCC stigma toward people with OUD. This study examined whether an online training incorporating patient narratives reduced PCCs’ stigma toward people with OUD (primary) and increased intentions to treat people with OUD compared to an attention-control training (secondary).

**Methods:**

PCCs from 15 primary care clinics were invited to complete a 30 min online training for an electronic health record-embedded clinical decision support (CDS) tool that alerts PCCs to screen, diagnose, and treat people with OUD. PCCs were randomized to receive a stigma-reduction version of the training with patient narrative videos or a control training without patient narratives and were blinded to group assignment. Immediately after the training, PCCs completed surveys of stigma towards people with OUD and intentions and willingness to treat OUD. CDS tool use was monitored for 6 months. Analyses included independent samples *t*-tests, Pearson correlations, and logistic regression.

**Results:**

A total of 162 PCCs were randomized; 88 PCCs (58% female; 68% white) completed the training (Stigma = 48; Control = 40) and were included in analyses. There was no significant difference between intervention and control groups for stigma (*t* = − 0.48, *p* = .64, Cohen’s *d* = − 0.11), intention to get waivered (*t* = 1.11, *p* = .27, *d* = 0.26), or intention to prescribe buprenorphine if a waiver were no longer required (*t* = 0.90, *p* = 0.37, *d* = 0.21). PCCs who reported greater stigma reported lower intentions both to get waivered (*r* = − 0.25, *p* = 0.03) and to prescribe buprenorphine with no waiver (*r* = − 0.25, *p* = 0.03). Intervention group and self-reported stigma were not significantly related to CDS tool use.

**Conclusions:**

Stigma toward people with OUD may require more robust intervention than this brief training was able to accomplish. However, stigma was related to lower intentions to treat people with OUD, suggesting stigma acts as a barrier to care. Future work should identify effective interventions to reduce stigma among PCCs.

*Trial Registration*: ClinicalTrials.gov NCT04867382. Registered 30 April 2021—Retrospectively registered, https://clinicaltrials.gov/ct2/show/NCT04867382

## Introduction

Nearly three-quarters of the more than 91,000 drug overdose deaths in the United States in 2020 involved an opioid, representing a 37% increase in opioid-related deaths from 2019 [1]. Given the rise in opioid-related deaths, increasing the availability and uptake of efficacious treatments, including medications for OUD (MOUDs), is critical. However, only 20% of patients diagnosed with OUD seek treatment, and only 25% of those receive MOUDs [2]. MOUDs, including buprenorphine and naltrexone, can be used to treat OUD in primary care settings, which could widely increase the availability of treatment [3]. However, there is a shortage of clinicians waivered to prescribe buprenorphine, and less than one-third of the clinicians waivered to prescribe buprenorphine do so [4].

One barrier to scaling up MOUD treatment in primary care is stigma toward people with OUD. Health-related stigmas are social processes by which people are labeled, stereotyped, devalued, and rejected because they have a health condition [5]. Such stigmas occur when a person perceives a difference between themselves and the person with the health condition, which leads to disdain for that person [6, 7]. People with substance use disorders (SUDs) are more stigmatized than people with other conditions, including mental illness and physical disabilities [8, 9]. Common stereotypes about people with SUDs include that they are responsible for their condition (and could control their substance use), dangerous, unpredictable, unemployable, and criminals [10–12]. Indeed, substance use is often viewed as a moral and criminal issue, rather than a chronic illness [11]. Despite the clear implications of the impact of stigma on people with SUDs, when compared to the large literature base on methods to address stigma for mental health conditions, [6, 13, 14] there is relatively little empirical literature on effective ways to reduce stigma for SUDs in general [10] and OUD in particular [15].

Stigma is considered a “major driver” behind the lack of access to MOUDs, because of the misconception that addiction is a volitional choice, the separation of addiction treatment from the rest of the medical system, and the language used for addiction (e.g., calling urine drug screens “clean” or “dirty” or patients “addicts” or “junkies”) [16, 17]. Offering MOUDs in primary care has the potential to reduce stigma and to increase the likelihood that patients will seek treatment [18, 19]. Although there is a dearth of research on PCC stigma toward people with OUD, a study of more than 1000 physicians found they hold many of the same stigma beliefs as the general public [20]. Clinician stigma may also lead to withholding of primary care [21, 22] and pharmacy services [23, 24] from patients with SUDs, especially among those who inject drugs [25].

There are few evidence-based interventions for reducing OUD stigma. Common approaches to reducing health-related stigma in the general public are to provide education, facilitate contact between persons affected by the condition and members of the general public, and target popular opinion leaders or change agents [26, 27]. Systematic reviews indicate stigma interventions for healthcare clinicians vary in content and mode of delivery, but generally use a combination of education and direct contact with people with SUDs. These reviews conclude that improving attitudes about people with SUDs may be best achieved through communication strategies that promote positive stories and demonstrate that stigmatized characteristics are not representative of all people in a stereotyped group [28]. Others have suggested that combining personal narratives from individuals being treated with MOUDs with science-based education about OUD and the benefit of MOUDs could be particularly effective [16]. Additionally, clinicians can learn to “disentangle behavior from identity” by adopting language that frames addiction as a treatable health condition and not a personal failing [29]. Importantly, to our knowledge, there are no evidence-based interventions designed to reduce stigma among practicing PCCs.

This study examined whether an intervention designed to reduce PCC stigma of persons with OUD, compared to an attention-control training, was related to stigma, intentions to get waivered to prescribe buprenorphine or to prescribe buprenorphine if a waiver were no longer required, and use of a clinical decision support (CDS) tool embedded in the electronic health record (EHR) designed to help PCCs screen, diagnose, and treat people with OUD. We also examined secondary outcome measures, including reported willingness to work with people with OUD, perceived treatment effectiveness, and perceived likelihood that patients would adhere to OUD treatment. We hypothesized that the intervention incorporating patient narratives would reduce stigma, increase intentions to get waivered to or prescribe buprenorphine, and increase CDS tool use. In secondary hypotheses, we hypothesized that the stigma reduction intervention would be associated with increased willingness to work with people with OUD, increased perceptions that the treatments were effective, and greater perceptions that people with OUD would adhere to the treatments. As a second aim, we examined whether PCC self-reported stigma was related to intention to get waivered or prescribe buprenorphine and to likelihood of using the CDS tool. We hypothesized that PCCs with lower stigma toward people with OUD would have greater intentions to get waivered or prescribe buprenorphine and would be more likely to use the CDS tool. In secondary analyses, we hypothesized that PCCs with greater stigma would also report lower willingness to work with people with OUD, that treatments would be less effective, and that people would be less likely to adhere to the treatments.

## Methods

### Study setting

This study was conducted at HealthPartners, a nonprofit healthcare organization that cares for more than 1.2 million patients in Minnesota and western Wisconsin. Approximately 7% of HealthPartners PCCs are waivered to prescribe buprenorphine. Fifteen clinics were given access to an CDS tool to identify, screen, diagnosis, and treat OUD; PCCs in those clinics were invited to participate in this study. The HealthPartners IRB reviewed and approved this study.

### Study design

This study used a randomized controlled trial (RCT) design. PCCs were randomized to receive one of two trainings via an online learning platform: a stigma reduction training or an attention-control training (ClinicalTrials.gov NCT04867382). PCCs completed outcome surveys immediately following the training in REDCap [30]. Additionally, EHR data were extracted in the 6 months following the training to measure PCC use of a CDS tool for OUD.

### Participants

Participants were PCCs who had access to the CDS tool as part of an ongoing pragmatic clinical trial examining the impact of the CDS tool on OUD patient care [31]. PCCs were eligible for this study if they practiced in one of the CDS tool intervention clinics and they were a family physician or general internist (MD/DO) or adult-care non-obstetric nurse practitioner (NP) or physician assistant (PA). Eligible PCCs (*N* = 162) across 15 clinics were invited to participate in the study via emails from the online learning platform. PCCs provided informed consent before beginning the training.

### Randomization

Stratified randomization with one constrained covariate was used to assign PCCs 1:1 to the stigma reduction or attention-control training group. The intervention clinic at which the PCC practiced was a stratifying variable, and PCC waiver status (waivered, not waivered) was balanced study-wide across training groups. The study statistician (ALC) generated the randomization assignment using SAS software prior to PCCs being invited to complete the training. PCCs were blind to training assignment.

### Trainings

#### Intervention delivery

Both the stigma reduction intervention and the attention-control trainings were delivered via an online learning platform and took 25–35 min to complete.

#### Common intervention components in both trainings

General training in both groups included science-based education about OUD and MOUDs and how to use the CDS tool, including how to review relevant chart history, make a diagnosis, choose a treatment option, conduct follow-up, and manage comorbid conditions. PCCs were presented with four prototype patients at risk for or with OUD to demonstrate aspects of the CDS tool.

#### Stigma reduction training

Contact with people sharing personal narratives (including a story of “on the way down” and “on the way up”) is the most powerful intervention to reduce public stigma of health conditions [13, 32]. For this study, we adapted this approach showing video vignettes of patient narratives and demonstrations of non-stigmatizing language. Our team worked with a medical historian who had interviewed people with OUD to compile four realistic stories of people with OUD; we created scripts of patient-PCC interactions to demonstrate various aspects of the tool and to insert patient narratives. Professional actors were filmed as “patients” telling their stories, and videos were embedded in the training. For example, one narrative was the story of a patient who developed OUD after taking opioids for a severe sore throat. PCCs viewed the training with the embedded video of the “patient” (actor) telling their story, and the trainer walked through how to use the CDS tool. The short narratives provided de-stigmatizing context and demonstrated CDS tool applicability to diverse populations.

#### Attention-control training

The attention-control training included the general components described above but omitted the stigma reduction component (patient narrative videos). This comparison training controlled for attention, contact time and extra training on using CDS tool [33]. Participants saw the same cases that were presented in the stigma reduction training (e.g., OUD following treatment of a severe sore throat), but they did not see patient videos or hear narratives. Instead, they saw the EHR and the trainer talking about the cases in generalities (e.g., “This is a patient who is at high risk for OUD.”).

#### Procedure

Eligible PCCs received the invitation and up to three reminder emails to join the study. The training period was between April and May 2021. Immediately after completing the training, PCCs completed a survey via REDCap, [30] a secure data management software, that included self-report outcome measures listed below. PCCs were given $100 gift cards as compensation. PCC prescribing and referral behavior in the 6 months prior to the training and CDS tool use data in the 6 months following the training were extracted from the EHR.

### Primary outcome measures

#### OUD stigma

The Difference, Disdain, and Blame scales measured stigma toward people with OUD [6, 7, 34]. We chose this measure because people are more willing to state that people with a health condition are different from them than to endorse general stigmatizing beliefs, therefore reducing social desirability in stigma measurement [6]. Three items measured difference (people with OUD are not similar, like, or comparable to others), three items measured disdain (people with OUD are not good, respected, or favorable compared to others), and three items measure blame (people with OUD are responsible for their condition) [14, 35]. Items are scored on a 9-point agreement scale; some items are reverse-scored so that higher scores reflect greater stigma. Evidence suggests that the scales demonstrate good internal consistency [7] and are positively associated [15]. In our sample, one item on the blame subscale was poorly correlated with the other items and affected internal consistency (“How blamed do you think people with opioid use disorder are for their illness?”); thus, this item was omitted. Internal consistency of the overall 8-item scale (α = 0.80) and the difference subscale was high (α = 0.87); internal consistency of the disdain (α = 0.70) and blame (α = 0.60) subscales was acceptable.

#### Intentions to get waivered to prescribe buprenorphine

Non-waivered PCCs rated one question on their intention to get waivered to prescribe buprenorphine (“How likely are you to get waivered to prescribe buprenorphine in the next year?”) on a five-point Likert-type scale ranging from 1 (*I definitely will not*) to 5 (*I definitely will*).

#### Intentions to prescribe buprenorphine should a waiver no longer be required

This study was conducted just as the federal government was deliberating eliminating the training requirement to get a waiver to prescribe buprenorphine. We, therefore, added a question regarding this potential scenario. Non-waivered PCCs rated one question on their intentions to prescribe buprenorphine if a waiver were no longer required (“If your patient with OUD requested buprenorphine in the next year and a waiver were no longer required, would you prescribe buprenorphine?”) on a five-point Likert-type scale ranging from 1 (*I definitely would not*) to 5 (*I definitely would*).

#### CDS tool use

PCC CDS tool use was defined as clicking within the CDS tool, such as screening for OUD, making a diagnosis, providing a referral, prescribing a medication, printing patient education materials, or prescribing naloxone. The use rates for each PCC for the follow-up period (6 months) were calculated as the proportion of CDS -eligible visits in which the CDS tool was clicked. An CDS -eligible visit was defined as a primary care visit with a patient between 18 and 75 who had either (1) a diagnosis of OUD, (2) an opioid overdose within the prior 6 months, (3) a prescription for a MOUD (buprenorphine, methadone, or IM naltrexone), or (4) high risk for OUD or overdose, determined by a risk prediction algorithm embedded in Epic, defined as a score of 55 or higher (out of 100) at the time of the encounter [36]. The CDS tool is designed for both waivered and non-waivered PCCs, and guides them through screening, diagnosis, and treatment (either referral or prescription). Because overall use rates were low, this variable was dichotomized as whether the PCC ever clicked in the tool (1 = yes; 0 = no) in the 6 months following the training.

### Secondary outcome measures

#### Willingness to work with people with OUD

PCCs reported their willingness to work with people with OUD using 3 items adapted from the Drug Problems Perceptions Questionnaire [37]. PCCs responded to each item using a 5-point Likert-type scale ranging from 1 (*strongly disagree*) to 5 (*strongly agree*). One item is reverse-scored (“I would enjoy my job more if I could stop working with patients with opioid use disorder”) and the items were averaged for a total score, with higher scores corresponding to greater willingness to work with people with OUD.

#### Opioid treatment outcome expectancies

Treatment outcome expectancies were assessed using two items developed for this study. First, PCCs rated the extent to which they believe available treatments are effective for treating OUD on a 4-point Likert-type scale ranging from 1 (*not at all effective*) to 4 (*very effective*). The second item asked PCCs to rate the extent they believed patients would adhere to those treatments on a 4-point Likert-type scale ranging from 1 (*not at all likely*) to 4 (*very likely*). Items were analysed separately.

#### Power analysis

In our a priori power analysis, we estimated that with a sample of 112 PCCs, α = 0.05, and 80% power, we would be able to detect a moderate-sized difference in stigma (Cohen’s *d* = 0.49) and intentions to get waivered (*d* = 0.55) between the groups. Further, with a CDS tool use rate of 30% eligible encounters, we would be able to detect a relative risk difference of 1.58.

#### Data analysis

Descriptive statistics were used to examine the central tendency and variability of measures across and between groups. Independent samples *t*-tests were used to compare stigma reduction and attention-control groups on self-reported outcome measures (i.e., stigma and intentions to get waivered or prescribe buprenorphine if a waiver were not required). Cohen’s *d* was calculated as a measure of effect size. Firth regression was used to predict whether a PCC used the CDS tool (1 = used, 0 = did not use) in the 6 months following the training, with training group as the independent variable. The Firth penalized likelihood approach was used to partially correct for small sample bias [38].

For the second aim, Pearson correlations were used to examine the relationship among self-reported stigma, intentions to get waivered and prescribe buprenorphine should a waiver no longer be required, willingness to work with people with OUD, and perceived effectiveness of and adherence to OUD treatment. To examine the relationship between stigma and CDS tool use, Firth regression was used to predict whether a PCC used the CDS tool in the 6 months following the training, with stigma as the independent variable.

## Results

**Fig. 1:**
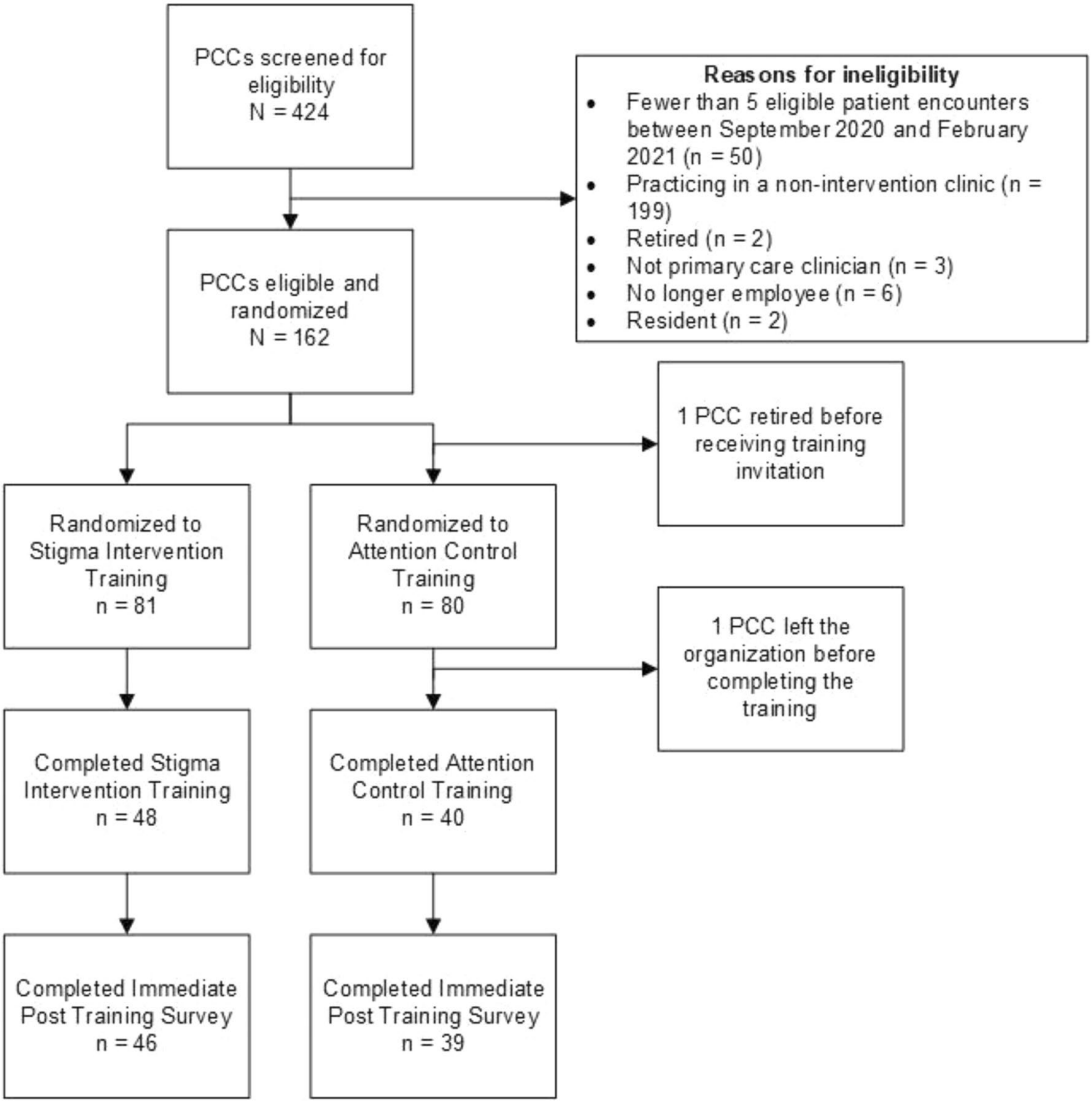
Study flow diagram

Of the 162 PCCs invited to participate, 88 PCCs completed the training, and 85 completed the immediate post-training survey (see Fig. 1). We examined whether there were pre-randomization differences in known characteristics of the PCCs who completed the training relative to those who did not. Advanced practice clinicians were significantly more likely than physicians to complete the training (76.9% v. 47.9%; *OR* = 3.62 (95% CI = 1.58, 8.27), *p* < 0.01), and PCCs from metro-based clinics were somewhat more likely than PCCs practicing in rural clinics to complete the training (58.3% v. 33.3%; *OR* = 2.79 (95% CI = 1.06, 7.35), *p* < 0.05). There were no differences in completion by waiver status (*OR* = 1.10 [95% CI = 0.36, 3.33], ns).

Demographic characteristics of participating PCCs overall and by treatment group are presented in Table 1. Clinicians were, on average, 47.4 years old (*SD* = 11.6; range = 29–70 years). PCCs who participated in the study infrequently treated patients with OUD prior to the training. In the 6 months prior to the intervention, 2.4% had made at least one referral to addiction medicine, 11.9% had made at least one referral to pain management, and none had ever referred to a waivered PCC. Waivered clinicians (*n* = 8) prescribed a MOUD at an average of 4.5 visits (range 0–12) in the 6 months prior to the study.

**Table 1:** Demographic characteristics of PCCs who completed stigma reduction or attention-control training

	All *N* = 85		Stigma reduction *n* = 46		Attention-control *n* = 39	
Gender						
Male	31	36.5%	17	37.0%	14	36.0%
Female	49	57.7%	27	58.7%	22	56.4%
Not listed	1	1.2%	1	2.2%	0	0.0%
Prefer not to answer	4	4.7%	1	2.2%	3	7.7%
Ethnicity						
Hispanic or Latino	3	3.5%	1	2.2%	2	5.1%
Not Hispanic or Latino	80	94.1%	45	97.8%	35	89.7%
Missing	2	2.4%	0	0%	2	5.1%
Race						
Asian	8	9.4%	6	13.0%	2	5.1%
Black or African American	6	7.1%	3	6.5%	3	7.7%
White	57	67.1%	29	63.0%	28	71.8%
Multiple or Other	2	2.4%	1	2.2%	1	2.5%
Prefer not to answer	12	14.1%	7	15.2%	5	12.8%
Waivered to prescribe buprenorphine	8	9.4%	3	6.5%	5	12.8%
Degree						
MD/DO	55	64.7%	30	65.2%	25	64.1%
PA/NP	30	35.3%	16	34.8%	14	35.9%

*MD/DO* Doctor of Medicine or Doctor of Osteopathic Medicine, *PA* Physician Assistant, *NP* Nurse Practitioner

There were no significant differences between the stigma reduction training and attention-control groups on self-reported stigma, intentions to get waivered, or intentions to prescribe buprenorphine if a waiver were no longer required immediately following the training (see Table 2). In the 6 months following the training, 82 of the 88 PCCs had at least one eligible CDS tool visit. Of those with eligible visits, 7 PCCs used the tool at least once (Stigma Reduction, *n* = 3 [6.8%]; Attention-Control, *n* = 4 [10.5%]). There was no significant difference between the groups in the likelihood that they used the CDS tool in the 6 months following training, *OR* = 0.65 (95% CI = 0.15, 2.86), *p* = 0.57. Stigma was not significantly related to PCC degree (physician vs. advanced practice clinician; *p* = 0.95) or to waiver status (*p* = 0.98).

**Table 2:** Effect of stigma reduction v. attention-control training on self-reported stigma and intentions to treat people with OUD

	Stigma reduction *M (SD)*	Attention-control *M (SD)*	*t*	*p*	Cohen’s *d*
Overall stigma	4.1 (1.3)	4.3 (1.2)	− 0.48	0.63	− 0.11
Difference	3.4 (1.8)	3.1 (1.7)	0.74	0.46	0.16
Disdain	4.7 (1.4)	4.9 (1.4)	− 0.86	0.39	− 0.19
Blame	4.4 (1.6)	4.8 (1.6)	− 1.29	0.20	− 0.28
Intentions to get waivered	2.3 (0.7)	2.1 (0.8)	1.11	0.27	0.26
Intentions to prescribe buprenorphine	3.2 (1.0)	3.0 (0.9)	0.90	0.37	0.21
Willingness to work with OUD	3.0 (0.7)	3.1 (0.9)	− 0.83	0.41	− 0.18
Perceived OUD treatment effectiveness	2.6 (0.8)	2.7 (0.7)	− 0.74	0.46	− 0.16
Perceived OUD treatment adherence	2.5 (0.6)	2.4 (0.6)	0.15	0.88	0.03

In a second aim, we examined whether stigma was related to intentions to get waivered, intentions to prescribe buprenorphine if a waiver were no longer required, willingness to work with OUD, and perceived treatment efficacy and compliance (see Table 3). PCCs who reported greater stigma toward people with OUD also reported lower intentions to get waivered or prescribe buprenorphine if a waiver were not required, less willingness to work with people with OUD, and lower perceived treatment efficacy and compliance. Stigma was not significantly related to likelihood of using the CDS tool in the 6 months following the training, *OR* = 1.75 (95% CI = 0.86, 3.56), *p* = 0.12.

**Table 3:** Correlations among Self-Reported Stigma and Intentions to Treat People with OUD (N = 85)

	1	2	3	4	5	6	7	8
1. Stigma	–							
2. Difference	0.84^c^	–						
3. Disdain	0.79^c^	0.47^c^	–					
4. Blame	0.64^c^	0.31^b^	0.33^b^	–				
5. Willingness to work with OUD	− 0.40^c^	− 0.34^b^	− 0.29^b^	− 0.34^b^	–			
6. Intention to get waivered	− 0.25^a^	− 0.06	− 0.23^a^	− 0.35^b^	0.42^c^	–		
7. Intention to prescribe buprenorphine	− 0.25^a^	− 0.11	− 0.18	− 0.35^b^	0.46^c^	0.62^c^	–	
8. Perceived OUD treatment effectiveness	− 0.32^b^	− 0.19	− 0.26^a^	− 0.38^c^	0.46^c^	0.21	0.29^a^	–
9. Perceived OUD treatment adherence	− 0.39^c^	− 0.28^a^	− 0.31^b^	− 0.35^b^	0.36^c^	0.17	0.22	0.62^c^

^a^p < .05

^b^p < .01

^c^p < .001

## Discussion

This study demonstrated that a stigma reduction intervention was not significantly related to PCC stigma, intentions, or CDS tool use. However, as hypothesized, PCCs who reported greater stigma also had lower desire to work with patients with OUD (across multiple measures, including intentions to get waivered, intentions to prescribe buprenorphine, and willingness to work with OUD) and believed that treatments were less effective and patients would be less likely to adhere to treatments. However, stigma was not related to an objective measure of management of OUD, i.e., CDS tool use. Thus, stigma likely acts as a barrier to care for OUD in primary care settings based on PCC’s attitudes towards working with people with OUD, but it is not clear how this affects PCC behavior.

To our knowledge, only two interventions have been studied to reduce stigma toward patients with SUDs among practicing healthcare clinicians, [39, 40] and both targeted SUD in general, not OUD specifically. Further, both used quasi-experimental designs without a control group. Thus, this is one of the first studies to examine the impact of an intervention to reduce stigma toward people with OUD among PCCs using a RCT design. There may be several reasons why the stigma reduction training did not impact stigma and attitudes. First, the mode of delivery was a brief, online training. The benefits to this mode of delivery are that PCCs frequently complete online trainings similar to this intervention and it allows the intervention to be scaled up and delivered to large groups of PCCs with little to no extra cost after the training development. Nevertheless, the research team has no way to control for PCC engagement with the content to ensure treatment fidelity. Further, the training may have been too brief (25–35 min) to have a large effect. Interventions that have been effective in reducing stigma often involve repeated interactions with people with OUD [26, 27]. Given the job demands on PCCs, it may be difficult to encourage repeated engagement with training on one specific topic (e.g., OUD).

The stigma reduction training incorporated patient narratives to reduce stigma. Hearing patients tell their stories has been shown to be the most effective approach for reducing stigma towards people with a stigmatized health condition [28]. However, this approach may have been too subtle, and a more overt approach (e.g., discussing the impact of stigma, training PCCs to become aware of their own biases) combined with the patient narratives may be more effective. Moreover, although the patient narratives in the videos were based on real life experiences of people with OUD, the patients were actors. It may be that this did not translate into the voice of someone with lived experience, which could be a more powerful approach. Finally, we chose to compare the stigma reduction version of the training to the same training without a stigma component. This is a strong comparator (controls for time and attention), [33] and offering science-based education about OUD and treatments may have served as an intervention. We were not able to collect surveys from PCCs who did not complete the training on stigma towards people with OUD. Further, we chose not to include a baseline (pre-training) measure among the PCCs to avoid hinting to PCCs that stigma was a primary outcome. Measuring stigma is challenging because of social desirability bias; therefore, we restricted measurement to one time point. These design choices limited our ability to determine if both trainings reduced stigma or if PCCs who completed the training had lower stigma at the outset.

Our hypothesis that stigma towards OUD was related to desire to treat OUD was supported. Specifically, PCCs who reported more stigma also reported lower intentions to get waivered and to prescribe buprenorphine if a waiver were no longer required and less willingness to work with patients with OUD. Further, stigma was related to their perceived effectiveness of and patients’ adherence to treatment for OUD. Previous studies have shown that healthcare providers hold many of the same stigmatizing beliefs toward people with OUD as the general population [20, 41]. Our study suggests that PCC stigma may also impact the likelihood that they would be willing to treat someone with OUD, which may limit access to care to treatment in primary care settings. Increasing the availability of OUD treatment in primary care has been a major goal to reduce barriers to care; however, our data and others’ [42] suggest PCC stigma may also need to be addressed to increase availability of treatment.

Our study also examined whether training assignment and self-reported stigma were related to an objective measure of PCC behavior: CDS tool use in the 6 months following the training. A major limitation of this analysis was that PCCs infrequently clicked into the CDS tool. Although PCCs in our system have used CDS tools in our system before, [43] the OUD CDS tool is one of the first that encourages PCCs to click in a tool in the EHR (previously they were only encouraged to review printed materials). The small number of PCCs who clicked into the tool limited our ability to examine group differences and correlations. It may be that PCCs used the paper version of the CDS tool or were evaluating and treating OUD without using the CDS tool; unfortunately, these actions are not reliably measured in the EHR and were not included in our assessment of tool use.

The strengths of this study include the randomized design and the delivery of an intervention to practicing PCCs. In addition to the design limitations described previously, there was no measure of training engagement or treatment fidelity, and the intervention was delivered in one integrated healthcare system, which may limit generalizability to other systems and settings. In addition, as noted in our a priori power analysis, we had goal of 112 PCCs completing the training. We had initially estimated that there would be more eligible PCCs in the clinics, and we were unable to encourage more than 50% of PCCs to participate, likely at least in part because this intervention was delivered during the COVID-19 pandemic and PCCs were understandably overwhelmed. Nonetheless, the observed effect sizes were very small, indicating that even with a larger sample size, we may not have seen statistically significant effects of our intervention. Finally, the measure of stigma was self-report, which may be subject to social desirability.

## Conclusion

This study demonstrated that a brief intervention to reduce PCC stigma towards people with OUD did not affect PCC stigma, intentions to treat people with OUD, or CDS tool use compared to an attention-control training. Given the findings that PCC stigma is related to intentions and willingness to treat people with OUD, finding effective stigma reduction interventions for this group is needed. Future interventions may consider including more educational and skills-based components (e.g., including information about the damaging effects of stigma and how to identify and combat one’s own biases) and repeated intervention exposure.

## Data Availability

The datasets used and/or analyzed during the current study are available from the corresponding author upon request.

## Abbreviations

EHR: Electronic health record
CDS: Clinical decision support
OUD: Opioid use disorder
PCC: Primary care clinician
MD/DO: Physician (doctor of medicine or doctor of osteopathic medicine)
MOUD: Medication for opioid use disorder
NP: Nurse practitioner
PA: Physician assistant
RCT: Randomized controlled trial
SUD: Substance use disorder
